# The utilization of colonoscopy in Germany

**DOI:** 10.17886/RKI-GBE-2017-126

**Published:** 2017-12-13

**Authors:** Anne Starker, Nina Buttmann-Schweiger, Klaus Kraywinkel, Ronny Kuhnert

**Affiliations:** Robert Koch Institute, Department of Epidemiology and Health Monitoring, Berlin

**Keywords:** COLORECTAL CANCER, COLONOSCOPY, CANCER SCREENING, HEALTH MONITORING, GERMANY

## Abstract

Colorectal cancer is one of the three most common cancers in German adults. There are several legally based examinations for the early detection of colorectal cancer. People aged 50 to 54 years can have a faecal blood test annually. From the age of 55, this test is offered every two years as part of cancer screening, or alternatively a preventive colonoscopy, which can be repeated after ten years if the findings are inconspicuous. However, colonoscopies are also carried out to clarify complaints or other diseases (curative colonoscopy). In the GEDA 2014/2015-EHIS study, the respondents provided the date of their last colonoscopy. No data was collected on the reason why this colonoscopy was performed. 57% of women and 61% of men aged 55 years or older reported that they had a colonoscopy within the last 10 years. New legal regulations envisage the expansion of colorectal cancer screening and its development into an organised, quality-assured early detection programme.

## Introduction

Colorectal cancer is one of the three most common types of cancer among women and men in Germany; approximately one in eight new case of cancer affects the intestine. The risk of developing the disease increases steadily with age, and the majority of patients diagnosed with this condition are over 70 years old [1]. In 2015, every ninth death from cancer was attributable to cancer of the intestine. This makes colorectal cancer the third most common cause of death from cancer among men and women in Germany [2].

There are several legally based examinations for the early detection of colorectal cancer. According to the cancer screening guidelines, people aged 50 to 54 years can have a faecal blood test annually. Faecal blood can be an indication of intestinal polyps or even cancer and a positive result needs to be clarified by a colonoscopy. People aged 55 or over with a negative result can repeat the test as part of the cancer screening framework every two years [3]. As an alternative, people aged 55 or above are offered an early detection colonoscopy (preventive colonoscopy), which can be repeated after ten years if the test returns a negative result.

The general aim of cancer screening is to detect cancer and its precursors as early as possible so as to improve patients’ chances of recovery and their quality of life and therefore reduce cancer-specific disease frequency and mortality. Colonoscopy involves examining the entire length of the colon for proliferation of the intestinal mucosa, known as intestinal polyps, as well as the presence of potentially cancerous tissue. The intestinal polyps from which colorectal cancer can develop can usually be removed during the examination. In addition to the early detection of malignant tumours, colonoscopies can even help prevent colorectal cancer from developing at all. However, a colonoscopy is also performed to clarify symptoms and findings, such as visible blood in the stool, changes in bowel movements over several weeks or abdominal pain and cramps, which indicate an already existing (other) bowel disease. Colonoscopy is an elaborate procedure that is often viewed as unpleasant. However, it rarely results in complications that require treatment [4]. If complications do occur, they usually involve bleeding caused by the removal of intestinal polyps and only require outpatient treatment.

**GEDA 2014/2015-EHIS**

**Data holder:** Robert Koch Institute

**Aims:** To provide reliable information about the population’s health status, health-related behaviour and health care in Germany, with the possibility of a European comparison

**Method:** Questionnaires completed on paper or online

**Population:** People aged 18 years and above with permanent residency in Germany

**Sampling:** Registry office sample; randomly selected individuals from 301 communities in Germany were invited to participate

**Participants:** 24,016 people (13,144 women; 10,872 men)

**Response rate:** 26.9%

**Study period:** November 2014 - July 2015

**Data protection:** This study was undertaken in strict accordance with the data protection regulations set out in the German Federal Data Protection Act and was approved by the German Federal Commissioner for Data Protection and Freedom of Information. Participation in the study was voluntary. The participants were fully informed about the study’s aims and content, and about data protection. All participants provided written informed consent.

More information in German is available at www.geda-studie.de

Since 2003, there has been a decline in the number new cases of colorectal cancer in Germany. The age-standardised incidence rate, which takes into account the aging population, has fallen by about 18% among women and about 16% among men during this period [5]. This is seen as a positive result of colorectal cancer screening programme which was introduced in 2002. Nevertheless, 28,360 women and 34,050 were newly diagnosed with colorectal cancer in 2013 and 12,085 women and 13,608 men died from it [6].

## Indicator

In the GEDA 2014/2015-EHIS study collected data on the utilization of the last colonoscopy using a self-administered questionnaire which the respondents completed on paper or online. It included the question: ‘When was the last time you had colonoscopy?’ The following answers were accepted: ‘Within the past 12 months’, ‘1 to less than 5 years ago’, ‘5 to less than 10 years ago’, ‘10 years ago or more’ and ‘Never’.

This Fact sheet evaluates the utilization of the last colonoscopy undertaken within the last 10 years for women and men aged 55 and over. It is thus based on the recommendations for the cancer screening guidelines [3]. The results are stratified according to gender, age and level of education.

The analyses are based on data from 9,489 people (4,878 women; 4,611 men) aged 55 years or above at the time the survey was undertaken with valid data on the utilization of their last colonoscopy. All calculations were carried out using a weighting factor that corrects for deviations within the sample for the German population (as of 31 December 2014) with regard to gender, age, district type and level of education. The district type reflects the degree of urbanisation and accounts for the regional distribution in Germany. The International Standard Classification of Education (ISCED) was used to classify the educational and occupational qualifications [7]. A detailed description of GEDA 2014/2015-EHIS can be found in Lange et al. 2017 [8] as well as in the article German Health Update: New data for Germany and Europe in issue 1/2017 of the Journal of Health Monitoring.

## Results and discussion

According to GEDA 2014/2015-EHIS data, 56.6% of women and 60.8% of men in Germany aged 55 years or over reported that they had undergone a colonoscopy within the last 10 years (Table 1). Furthermore, less than half of women and men aged between 55 and 59 reported that they had undergone a colonoscopy in the last 10 years. There are no differences between woman and men in terms of educational levels. A higher proportion of men aged 70 years or above has undergone a colonos-copy within the last 10 years than women of the same age.

The proportion of people who reported having undergone a colonoscopy does not differ significantly between the federal states (data not shown).

A comparison of these results with those gained from the German Health Interview and Examination Survey for Adults (DEGS1, 2008-2011) shows that utilization has not changed significantly over time. DEGS1 found that 58.6% of women and 56.7% of men aged between 55 and 79 years reported having undergone a colonos-copy during the past 10 years [9]. The study also found no differences with regard to the utilization of colonos-copies going by socioeconomic status. As in GEDA 2014/2015-EHIS the reason for the colonoscopy were not asked.

Therefore, it is not possible to use this data to make valid conclusions about whether a colonoscopy was undertaken as part of cancer screening or due to the presence of relevant symptoms. In addition, it is unclear whether the examinations were carried out as part of outpatient treatment or during hospital stays. As such, other data sources are needed in order to estimate the utilization of early detection colonoscopy in Germany. Since the introduction of early detection colonoscopy in Germany in 2002, the Central Research Institute for Ambulatory Health Care in Germany (Zi) has been commissioned with undertaking scientific accompanying research. The institute provides estimates of utilization and assesses the quality of the examinations as well as the findings they produce. The data used by the institute comes from the routine documentation provided by the doctors who undertake the detection colonoscopies, which is sent to the Zi by the Association of Statutory Health Insurance Physicians (KV). These data demonstrate a cumulative rate of utilization of early detection colonoscopy among men and women aged 55 years or above of 20.8% for 2003 to 2014 [10]. This rate is significantly lower than the rate identified by GEDA 2014/2015-EHIS as the GEDA study only includes data on early detection colonoscopies carried out on an outpatient basis.

The data available from GEDA 2014/2015-EHIS provide a depiction of the utilization of colonoscopy in Germany. Both symptom-based (curative) colonoscopy and early-detection colonoscopy involve the same procedure – the removal of polyps (adenomas) in order to reduce the risk of colorectal cancer. From this it can be deduced that the relatively high prevalence of colonoscopy as a follow-up examination has also contributed to a reduced incidence of colorectal cancer in Germany, even though the effect cannot yet be quantified exactly.

The main risk factors for colorectal cancer are smoking and obesity but also those who exercise little, eat low-fiber diets and eat a lot of red meat or processed products are more likely to fall ill [4, 11]. However, the extent to which the risk of colorectal cancer can be reduced by changing the lifestyle is not certain [4].

In Germany, the 2008 implementation of the National Cancer Plan [12] led to a reorganisation of cancer screening. Moreover, the 2013 Cancer Screening and Registers Act established a legal basis with which to conceptually develop the early detection of colorectal cancer into an organised, quality-assured screening programme [13]. In particular, the eligibility criteria, the invitation procedure and the information to be provided to patients are to be revised. The invitation to screening and the information provided to patients are intended to assist people who are eligible for screening to decide whether they want to take part. The explicit aim is to provide more comprehensive information on the early detection of colorectal cancer and to promote the formation of an informed decision on participation [14]. It has already been realised that the fecal occult blood test has been carried out since January 2017 with a quantitative immunological test (i-FOBT) [3]. The systematic collection, monitoring and improvement of the quality of the structure, process and outcome quality of colorectal cancer screening, which is aimed for in the screening program, allows a more precise estimation of the effect of early detection colonoscopy on colorectal cancer incidence and mortality to be expected.

## Key statements

- 57% of women and 61% of men aged 55 years or older reported that they had a colonoscopy within the last 10 years.
- Colonoscopies are less frequently reported by women and men under the age of 60 years than by older age groups.
- In the 70-year-old and elderly age group, significantly more men than women report a colonoscopy.

## Figures and Tables

**Table 1:** The utilization of the last colonoscopy within the last 10 years among people aged 55 years or over by sex, age and educational level (n=4,878 women; n=4,611 men) Source: GEDA 2014/2015-EHIS

Women	%	(95% CI)	Men	%	(95% CI)
**Women (total)**	**56.5**	**(54.7-58.2)**	**Men (total)**	**60.8**	**(59.1-62.5)**
**55-59 Years**	42.8	(39.5-46.2)	**55-59 Years**	48.5	(44.7-52.2)
Low education	43.6	(35.1-52.4)	Low education	52.5	(41.6-63.2)
Medium education	41.9	(37.4-46.5)	Medium education	48.2	(42.6-53.8)
High education	45.5	(39.3-51.5)	High education	46.8	(41.5-52.2)
**60-64 Years**	57.4	(53.9-60.9)	**60-64 Years**	58.5	(54.7-62.2)
Low education	54.6	(46.6-62.3)	Low education	60.6	(48.7-71.3)
Medium education	56.9	(52.0-61.6)	Medium education	55.3	(49.3-61.1)
High education	62.7	(56.2-68.9)	High education	63.3	(57.8-68.8)
**65-69 Years**	64.6	(60.6-68.4)	**65-69 Years**	63.1	(59.0-66.9)
Low education	56.2	(48.3-63.8)	Low education	61.3	(50.5-71.0)
Medium education	68.1	(62.6-73.1)	Medium education	60.7	(54.5-66.5)
High education	65.3	(57.4-72.5)	High education	67.8	(62.5-72.6)
**≥70 Years**	59.1	(56.6-61.6)	**≥70 Years**	67.5	(65.0-70.0)
Low education	55.7	(51.7-59.7)	Low education	69.1	(64.0-73.7)
Medium education	61.8	(58.3-65.2)	Medium education	66.3	(62.5-69.9)
High education	64.0	(57.1-70.3)	High education	68.7	(64.5-72.6)
**Total (women and men)**	**58.5**	**(57.3-59.7)**	**Total (women and men)**	**58.5**	**(57.3-59.7)**

CI=confidence interval
